# Supplementary material for: Plasma Interleukin-6 Level Predicts the Risk of Arteriovenous Fistula Dysfunction in Patients Undergoing Maintenance Hemodialysis
Source: J Pers Med. 2023 Jan 12;13(1):151. doi: 10.3390/jpm13010151 (PMC9864732; doi:10.3390/jpm13010151)
Supplement: Supplementary file 1 [file jpm-13-00151-s001.zip › jpm-2140193-supplementary.pdf]

SUPPLEMENTAL APPLEDIX FOR THE STUDY:

Plasma interleukin-6 level predicts the risk of arteriovenous fistula dysfunction in patients undergoing maintenance hemodialysis

Supplementary Table 1. Baseline demographic and laboratory data of the study population according to the tertiles of plasma IL-6 level

	Tertiles of plasma IL-6 level			*p* value
	**Tertile 1 (n=98)**	**Tertile 2 (n=91)**	**Tertile 3 (n=93)**	
Age (year)	59.3 ± 12.9	59.8 ± 12.5	65.2 ± 12.0	0.002[e, f]
Male (n, %)	64 (65.3)	67 (73.6)	59 (63.4)	0.292
Body mass index (kg/m^2)	24.2 ± 6.2	23.6 ± 5.1	23.0 ± 5.3	0.327
Dialysis vintage (year) [a]	2.4 (1.1, 6.0)	3.5 (1.7, 6.6)	3.8 (1.6, 9.6)	0.054
Charlson Comorbidity Index	3.9 ± 1.5	4.0 ± 1.6	4.3 ± 1.5	0.150
Diabetes mellitus (n, %)	57 (58.2)	51 (56.0)	53 (57.0)	0.957
Previous history of CVD (n, %) [b]	89 (90.8)	79 (86.8)	86 (92.5)	0.419
Pre-dialysis systolic BP (mmHg)	145 ± 18	143 ± 20	145 ± 22	0.895
Location of AVF (n, %)[c]				
Forearm	20 (20.6)	21 (23.1)	28 (30.1)	0.291
Upper arm	77 (79.4)	70 (76.9)	65 (69.9)	
AVF vintage (year) [a]	2.2 (1.0, 5.0)	2.9 (1.2, 5.3)	2.9 (0.9, 5.8)	0.222
Ultrafiltration (L/session)	2.17 ± 1.06	2.42 ± 0.99	2.32 ± 1.07	0.250
Single pool Kt/V	1.61 ± 0.29	1.55 ± 0.27	1.60 ± 0.30	0.276
Blood flow rate (mL/min)	272 ± 23	265 ± 23	269 ± 22	0.059
Hemodialysis duration (hour)	3.90 ± 0.22	3.93 ± 0.18	3.93 ± 0.22	0.499
Hemodiafiltration (n, %)	18 (18.4)	22 (24.2)	30 (32.3)	0.084
Statin use (n, %)	43 (43.9)	46 (50.5)	42 (45.2)	0.626
Anti-platelet agent use (n, %)	66 (67.3)	67 (73.6)	68 (73.1)	0.566
Erythropoiesis-stimulating agent use (%)	93 (94.9)	80 (87.9)	85 (91.4)	0.228
Hemoglobin (g/dL)	10.6 ± 1.1	10.5 ± 1.2	10.2 ± 1.4	0.062
Intact parathyroid hormone (pg/dL)	241 ± 221	310 ± 256	286 ± 181	0.097
Calcium (mg/dL)	8.6 ± 0.8	8.5 ± 0.7	8.5 ± 0.9	0.506
Phosphorus (mg/dL)	4.8 ± 1.5	5.1 ± 1.3	4.6 ± 1.4	0.098
Albumin (mg/dL)	3.9 ± 0.3	3.9 ± 0.3	3.7 ± 0.3	<0.001[e, f]
Alkaline phosphatase (mg/dL)	96 ± 60	108 ± 75	129 ± 88	0.010[e]

LDL-cholesterol (mg/dL)	78 ± 25	77 ± 26	76 ± 26	0.897
Erythrocyte sedimentation rate (mm/hr)	23.7 ± 19.4	25.9 ± 15.0	40.5 ± 28.0	0.001[e, f]
hs-CRP (mg/dL) [a]	0.22 (0.06, 1.49)	0.69 (0.19, 2.82)	2.40 (0.60, 8.06)	<0.001[e, f]
MCP-1(pg/mL) [a]	162 (134, 205)	175 (141, 224)	162 (123, 226)	0.515
TNF-α (pg/mL) [a]	8.12 (5.33, 11.79)	10.92 (6.94, 13.61)	10.93 (7.01, 14.00)	0.002[d, e]

Abbreviation: AVF, arteriovenous fistula; BP, blood pressure; LDL, low-density lipoprotein; hsCRP, high-sensitivity C-reactive protein; IL-6: interleukin-6 ; MCP-1,monocyte chemoattractant protein-1 TNF-α: tumor necrosis factor-α.

[a] Data are expressed as median (first, third interquartile ranges) and are compared by the Kruskal-Wallis test because of their non-normal distributions.

[b] Includes congestive heart failure, myocardial infarction, coronary artery disease requiring percutaneous transluminal coronary angioplasty or coronary artery bypass surgery, ventricular arrhythmia, cardiac arrest, and sudden death.

[c] No information in a patient without AVF dysfunction.

[d] $p < 0.05$, tertile 1 vs. tertile 2; [e] $p < 0.05$, tertile 1 vs. tertile 3; [f] $p < 0.05$, tertile 2 vs. tertile 3.

Supplementary Figure 1. Correlation between plasma IL-6 levels and high sensitivity C-reactive protein

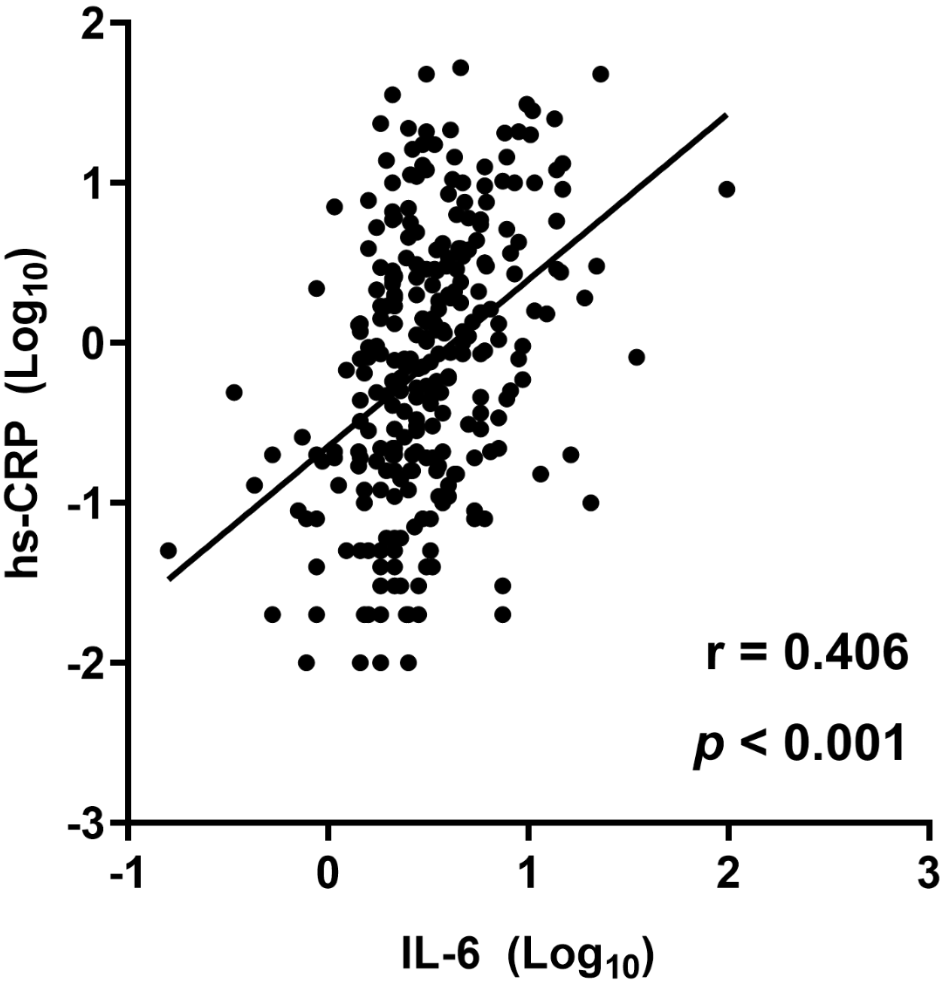

Abbreviation: IL-6, interleukin-6; hs-CRP, high sensitivity C-reactive protein.
